# The gene expression profile of preclinical autoimmune arthritis and its modulation by a tolerogenic disease-protective antigenic challenge

**DOI:** 10.1186/ar3457

**Published:** 2011-09-13

**Authors:** Hua Yu, Changwan Lu, Ming T Tan, Kamal D Moudgil

**Affiliations:** 1 Department of Microbiology and Immunology, University of Maryland School of Medicine, 685 West Baltimore Street, HSF-1, Suite 380, Baltimore, MD 21201, USA; 2 Department of Medicine, University of Maryland School of Medicine, 685 West Baltimore Street, MSTF-314, Baltimore, MD 21201, USA; 3 Division of Biostatistics and Bioinformatics, Department of Epidemiology and Public Health, University of Maryland School of Medicine, 685 West Baltimore Street, MSTF-261, Baltimore, MD 21201, USA; 4 Division of Rheumatology, Department of Medicine, University of Maryland School of Medicine, 685 West Baltimore Street, Baltimore, MD 21201, USA

**Keywords:** adjuvant arthritis, gene expression, heat shock proteins, immune tolerance, microarray analysis

## Abstract

**Introduction:**

Autoimmune inflammation is a characteristic feature of rheumatoid arthritis (RA) and other autoimmune diseases. In the natural course of human autoimmune diseases, it is rather difficult to pinpoint the precise timing of the initial event that triggers the cascade of pathogenic events that later culminate into clinically overt disease. Therefore, it is a challenge to examine the early preclinical events in these disorders. Animal models are an invaluable resource in this regard. Furthermore, considering the complex nature of the pathogenic immune events in arthritis, microarray analysis offers a versatile tool to define the dynamic patterns of gene expression during the disease course.

**Methods:**

In this study, we defined the profiles of gene expression at different phases of adjuvant arthritis (AA) in Lewis rats and compared them with those of antigen mycobacterial heat shock protein 65 (Bhsp65)-tolerized syngeneic rats. Purified total RNA (100 ng) extracted from the draining lymph node cells was used to generate biotin-labeled fragment cRNA, which was then hybridized with an oligonucleotide-based DNA microarray chip. Significance analysis of microarrays was used to compare gene expression levels between the two different groups by limiting the false discovery rate to < 5%. Some of the data were further analyzed using a fold change ≥2.0 as the cutoff. The gene expression of select genes was validated by quantitative real-time PCR.

**Results:**

Intriguingly, the most dramatic changes in gene expression in the draining lymphoid tissue *ex vivo *were observed at the preclinical (incubation) phase of the disease. The affected genes represented many of the known proteins that participate in the cellular immune response. Interestingly, the preclinical gene expression profile was significantly altered by a disease-modulating, antigen-based tolerogenic regimen. The changes mostly included upregulation of several genes, suggesting that immune tolerance suppressed disease by activating disease-regulating pathways. We identified a molecular signature comprising at least 12 arthritis-related genes altered by Bhsp65-induced tolerance.

**Conclusions:**

This is the first report of microarray analysis in the rat AA model. The results of this study not only advance our understanding of the early phase events in autoimmune arthritis but also help in identifying potential targets for the immunomodulation of RA.

## Introduction

Rheumatoid arthritis (RA) is a major global health problem that imposes a heavy socioeconomic burden on society [1,2]. The disease is characterized by chronic inflammation of the synovial joints, often leading to physical deformities [3,4]. The precise etiology of RA is not known. It is a multifactorial disease involving both genetic and environmental components [3,5,6]. The joint pathology results from concerted action of many different cell types (macrophages, T cells, B cells, fibroblasts, and so on) and diverse cellular and molecular pathways [3,4]. There is meager information about the early phase (preclinical) inflammatory and immune events that lead to the initiation of the disease process. There also is a need for reliable biomarkers of the disease, as well as new therapeutic agents with higher efficacy but less toxicity. Thus, there is an urgent need to comprehensively examine and define the complex pathogenesis of RA with the hope of identifying new targets for treatment as well as monitoring the disease process. However, the genetic heterogeneity of human populations and the limitation of obtaining preclinical (incubation phase) biological samples from RA patients pose formidable challenges. In this regard, experimental models of human RA offer an invaluable resource in examining some of the above-mentioned critical issues that cannot be directly addressed in RA patients.

Adjuvant-induced arthritis (AA) is a well-studied model of RA that has been used extensively to study the pathogenesis of RA as well as to test new, potentially antiarthritic compounds [7-12]. AA can be induced in the inbred Lewis (LEW) (RT.1^l^) rat by subcutaneous immunization with heat-killed *Mycobacterium tuberculosis *H37Ra (Mtb), and it shares several features with human RA [13,14]. Furthermore, different phases of arthritis (incubation, onset, peak and recovery) during the course of AA are clearly identifiable [15,16], making it a suitable model for the study of preclinical (incubation phase) events in the disease course. Because of the genetic homogeneity and controlled disease induction, AA is an appropriate model for examining early pathogenetic events of autoimmune arthritis and their modulation by therapeutic regimens such as immune-based approaches.

Antigen-induced tolerance is one of the immunomodulatory approaches actively being explored for the control of autoimmune diseases, including RA [17-20]. Studies by others [10-12,21] and us [22,23] in the AA model of RA have documented the efficacy of a variety of tolerogenic approaches for the prevention as well as the treatment of arthritis. For example, we have previously shown that tolerization of LEW rats with soluble mycobacterial heat shock protein 65 (Bhsp65), which represents one of the major disease-related antigens in AA, affords protection against subsequent induction of AA [22]. However, despite the significant advances in the field of immune tolerance [24], the molecular basis of the antiarthritic effects of a tolerogenic regimen is not yet fully defined. A system-wide analysis of the early phase events in arthritis and the molecular targets of an arthritis-protective tolerogenic regimen would significantly advance our understanding and management of the arthritogenic processes.

Microarray analysis offers a comprehensive tool with which to simultaneously examine thousands of genes relating to diverse pathways mediating biochemical, molecular, immunological and pathological events in the course of a disease. The readouts, consisting of increased, decreased or unchanged expression of a large panel of genes, offer insights into the concurrent changes in multiple interrelated pathways at a given time point in the healthy or diseased state. With the completion of the sequencing of the genomes of human, mouse and rat, the results of microarray analyses can be further extended to comparative analyses of homologous genes of interest. However, the early phase events are not easy to study in RA patients, and microarray gene expression profiling of rats with AA has not previously been reported. Therefore, we undertook this important and timely study of gene expression analysis in AA.

In this study, we examined the gene expression profiles of the draining lymph node cells (LNCs) of Mtb-immunized LEW rats and compared them with those of antigen (Bhsp65)-tolerized or antigen-naïve rats. The induction of AA in LEW rats following Mtb injection involves the priming of potentially pathogenic T cells within the draining lymph nodes [14,25-28], and these T cells then migrate into the target organ, the joints, to initiate the development of arthritis. Conceivably, there are dynamic alterations in the relative frequency and activity of arthritogenic vs. disease-regulating T-cell subsets within the draining lymph nodes during the disease course. Furthermore, the pathogenesis of arthritis involves not only lymphoid cells but also myeloid-lineage cells [29-31]. Therefore, to fully understand the expression of disease-relevant genes within the draining lymph nodes *in vivo *during the course of AA, we tested bulk LNCs instead of purified T cells alone.

We hypothesized that the early (incubation) period following Mtb injection of LEW rats is a critical phase of the disease (AA) during which the host immune system is modulated and steered toward arthritis induction. Furthermore, immune interventions such as antigen-induced tolerance, which prevent subsequent development of AA, would significantly influence the early phase molecular events. In this study, we first tested the unmodified *ex vivo *gene expression profiles at different phases of the disease (AA) in LEW rats. Thereafter we focused on the incubation phase of AA to determine the antigen (Bhsp65)-induced gene expression and how it is modulated by an immunomodulatory Bhsp65-induced tolerance approach. We identified a molecular signature of at least 12 differentially expressed genes (DEGs) that characterize the state of Bhsp65-induced tolerance. We believe that the results of our study will not only improve the attributes of the AA model *per se *but also provide useful insights into both the pathogenetic processes in RA and potential immunomodulatory targets for controlling this disease.

## Materials and methods

### Induction and evaluation of adjuvant arthritis

Male Lewis (LEW/SsNHsd) (LEW) (RT-1^1^) rats, five to six weeks old, were obtained from Harlan Sprague Dawley (Indianapolis, IN, USA) and housed in an accredited animal facility at the University of Maryland at Baltimore (UMB). All animal handling and experimental work was carried out in accordance with the National Institutes of Health guidelines for animal welfare, and the study was approved by the Institutional Animal Care and Use Committee at our institution. The animals were acclimated to the holding room for at least three days before the initiation of our experimental work. AA was induced in each LEW rat on day 0 by immunizing them subcutaneously at the base of the tail with 2 mg of heat-killed Mtb (Difco, Detroit, MI, USA) emulsified in 200 μL of mineral oil (Sigma-Aldrich, St Louis, MO, USA). The development of arthritis and its severity were evaluated regularly by examination of all four paws for signs of arthritis and graded on a scale from 0 to 4 per paw on the basis of redness, swelling and induration. Arthritis appeared by about days 10 to 12 after Mtb injection. The disease severity reached its peak by days 19 to 21, followed by spontaneous regression of inflammation. In this study, we selected specific time points in the course of AA that represent different phases, as follows: day 7, incubation (Inc) phase; day 21, peak (Pk) phase; and day 25, recovery (Rec) phase. Naïve (Nv) rats without any Mtb immunization served as the baseline controls. Three animals per group were killed at each of the above time points for LEW rats, and draining lymph nodes (superficial inguinal, paraaortic and popliteal) were harvested.

### Antigen-induced immune tolerance

LEW rats were injected intraperitoneally on alternate days with soluble Bhsp65 at a dose of 200 μg for a total of three injections [22]. Nine days after the first injection the rats were immunized subcutaneously with Mtb (day 0) for the induction of AA. These Bhsp65-tolerized, Mtb-immunized rats were killed at the Inc phase of AA, and their draining lymph nodes were harvested for further testing.

### Antigenic restimulation of lymph node cells *in vitro*

The draining LNCs of LEW rats (with or without the tolerogenic Bhsp65 pretreatment) were collected on day 7 after Mtb immunization. These LNCs were cultured at 37°C for 24 hours in a six-well plate (5 × 10^6 ^cells/well) in serum-free HL-1 medium (Lonza, Walkersville, MD, USA) with or without Bhsp65 (5 μg/ml). Thereafter the cells were processed for RNA extraction.

### Total RNA extraction and GeneChip hybridization

Total RNA was extracted from LNCs using TRIzol reagent (Invitrogen, Carlsbad, CA, USA) following the manufacturer's instructions. RNA was purified with the RNeasy Mini Kit (Qiagen Ltd, Crawley, UK). RNA concentration was determined spectrophotometrically (260/280 nm, 260/230 nm) using a NanoDrop ND-1000 spectrophotometer (NanoDrop Technologies/Thermo Scientific, Wilmington, DE, USA). The quality of RNA was further assessed using the RNA 6000 Nano LabChip Kit (Agilent Technologies Inc, Palo Alto, CA, USA) and the Agilent 2100 Bioanalyzer. The RNA integrity number (mean ± SD) of the RNA isolated from freshly harvested and unstimulated LNCs was 9.61 ± 0.26 with a coefficient of variation (CV) of 2.7%, whereas that of the RNA extracted from LNCs cultured *in vitro *with or without Bhsp65 was 8.0 ± 0.5 with a CV of 6.3%. Total RNA (100 ng) was used as the input for the amplification and generation of biotin-labeled fragment cRNA for expression analysis using the Affymetrix kit (Genechip WT Sense Target Labeling and Control Reagents) according to the protocol supplied by the manufacturer (Affymetrix Inc, Santa Clara, CA, USA). Labeled cRNA was hybridized with an oligonucleotide-based DNA microarray (GeneChip Rat Gene 1.0 ST Array; Affymetrix) for whole-transcript coverage analysis. This microarray platform contains 700,000 unique 25-mer oligonucleotide features (spots) representing 27,342 Entrez Gene IDs. Hybridization on GeneChip Fluidics Station 450, scanning and image processing on GeneChip Scanner 3000 7G and preliminary data management with Affymetrix Microarray Suite version 5.0 software (all manufactured by Affymetrix, Inc) were performed at the Genomics Core Facility at UMB in accordance with the manufacturer's guidelines.

### Microarray data analysis

Affymetrix.cel files were uploaded to the Affymetrix Expression Console™ 1.1, checked for quality and then corrected for background. The data were normalized, and the median was polished using a robust multiarray. All data were logarithmically transformed prior to statistical analysis. Thereafter significance analysis of microarrays was used to compare gene expression levels between two different groups (three independent experiments, that is, three chips per group, biological replicates) by limiting the false discovery rate (FDR) to < 5%. With this FDR, DEGs [32] were identified. Some of the data were further analyzed using a ≥2.0-fold change as the cutoff. A heat map showing changes in the expression levels (fold changes) of representative genes was generated in the R software program with the package "gplots." Specifically, the fold changes in expression levels derived from log_2 _scales were organized with Expression Profiler software using the average linkage hierarchical clustering method, with distance determined by the correlation. Further analysis was performed to identify the biological processes involving the DEGs using the UniProt databases [33]. Enrichment analysis [33] was performed on different features using the Gene Ontology (GO) and KEGG databases [34,35], which revealed themes indicative of inflammatory disease, immune response, antigen processing and presentation, and so on. The microarray experimental plan and data analysis in this study are in accordance with the MIAME (minimum information about a microarray experiment) guidelines [36]. The microarray data presented in this manuscript have been deposited in a public repository, the Gene Expression Omnibus (GEO) [GEO:GSE31314].

### Quantitative real-time polymerase chain reaction for measuring gene expression

RNA extracted from LNCs tested *ex vivo *or after *in vitro *restimulation was used to validate microarray data. Column-purified total RNA was reverse-transcribed using the iScript cDNA Synthesis Kit (Bio-Rad Laboratories, Hercules, CA, USA) with oligo(dT) primers as described by the manufacturer. cDNA templates for quantitative real-time PCR (qPCR) were prepared by diluting them 1:10 and then amplified by using specific primers (Sigma) in SYBR Green PCR Master Mix (AB Applied Biosystems, Warrington, UK) on the LightCycler Instrument (Roche Applied Science, Indianapolis, IN, USA). Expression of the following genes was analyzed: *IFN-γ, IL-10, IL-17, Nos2, Ccr5, Socs1 and Socs3*. The levels of mRNA were normalized to HPRT (hypoxanthine phophoribosyltransferase) controls. The cycle threshold (*C*_t_) values, corresponding to the PCR cycle number at which fluorescence emission reached a threshold above baseline emission, were determined, and the relative mRNA expression was calculated using the 2-^ΔΔ*C*t ^method [16]. The Bland-Altman method [37] was used to assess the agreement in gene expression obtained by using microarrays and qPCR for the selected genes.

## Results

### Gene expression profiles at different phases during the natural course of adjuvant arthritis in LEW rats

**Figure 1:**
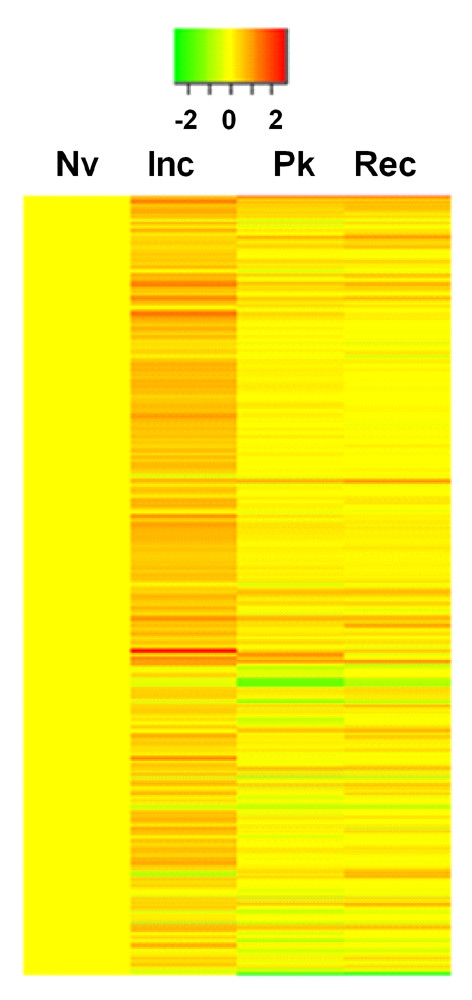
**Gene expression profiles of lymphoid cells *ex vivo *at different phases of adjuvant arthritis in Lewis rats**. Shown is a heat map representation of differentially expressed genes (DEGs) at the indicated phases of adjuvant arthritis (AA) in Lewis (LEW) (RT.1^l^) rats. Red denotes increased expression, and green indicates reduced expression compared to the baseline naïve (Nv) controls. At each phase of AA, total RNA was extracted from the draining *ex vivo *lymph node cells and tested using a microarray gene chip. Approximately 370 DEGs are shown. Inc, incubation; Pk, peak; Rec, recovery.

**Figure 2:**
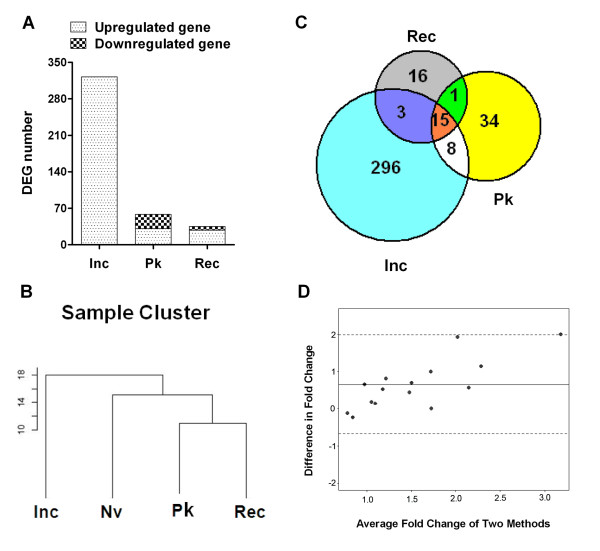
**Relationship of the differentially expressed genes at different phases of adjuvant arthritis**. **(A) **The number of differentially expressed genes (DEGs) at different phases of adjuvant arthritis (AA) compared to the rats in the naïve (Nv) state. **(B) **Dendrogram visualized using TreeView software [38] representing the relationship between DEGs at different phases of AA. **(C) **Venn diagram showing the number of DEGs and their overlap in the three data sets: Inc/Nv, Pk/Nv and Rec/Nv (Inc, incubation; Nv, naïve; Pk, peak; Rec, recovery). **(D) **Bland-Altman plots. The *x*-axis represents the average fold change of each sample measured by quantitative real-time PCR (qPCR) and microarrays. The *y*-axis is the difference in fold change calculated by qPCR measurements minus the microarray measurements for each sample. The solid line (*y *= 0.652) is the mean difference in fold change of all the samples. The two dotted lines represent two standard deviations away from the mean difference.

One of the goals of this study was to examine the *ex vivo *gene expression profiles of the draining LNCs of arthritic LEW rats at different phases of the disease, namely, the Inc, Pk and Rec phases. Nv LEW rats served as the baseline nonarthritic controls. The choice of *ex vivo *testing of LNCs was made to obtain a snapshot of the unperturbed gene expression profiles closely depicting the *in vivo *gene expression profiles. Three pairwise comparisons of gene expression patterns were performed: Inc-Nv, Pk-Nv and Rec-Nv. The results showed distinct gene expression profiles at different phases of AA (Figures 1 and 2A). As per our prediction, the most significant changes in gene expression were observed at the Inc phase, before the signs of arthritis appeared, instead of at the Pk phase of AA (Figures 1 and 2). This was evident by both the number and the level of expression of DEGs. Rats at the Inc phase had no overt signs of clinical arthritis (preclinical AA). A comparison of Inc rats with NV rats (Inc-Nv) revealed a relatively large number of DEGs. All DEGs (322 of the 29,214 screened probe sets) showed upregulation at the Inc period of AA compared to the baseline level (Figure 2A). In contrast, as described below, most of the genes were found to be downregulated during the Pk and Rec phases to the level of Nv rats (Figures 1 and 2A). Fewer genes (15 genes) maintained expression at a high level during the whole disease course (Inc through Rec phase). The major functional groups of DEGs at the Inc phase of AA are given in Tables 1 and 2.

**Table 1:** The major functional groups represented by 322 differentially expressed genes at the incubation phase of adjuvant arthritis in Lewis rats^a^

Functional group	Number (%)
Innate immunity	19 (5.9%)
Cell markers, innate immune response, complement	
Cell-mediated adaptive immune response and effector functions	23 (7.1%)
Stress protein-related and other autoantigens, antigen processing and presentation, T cell costimulation, cytokines and cytokine receptors, activators and regulators	
Humoral immunity	8 (2.5%)
Cell proliferation	113 (35.1%)
DNA synthesis, replication, repair, tRNA processing, transcription, translation, cell cycle and cellular components	
Cell migration	24 (7.4%)
Adhesion molecules, integrins, chemokines and receptors, cell migration-related	
Angiogenesis	2 (0.6%)
Oxygen metabolism related to pathogenesis of arthritis	11 (3.4%)
Transporters of oxygen, electrons, reactive oxygen species, cellular response to oxygen level and oxidation reduction	
Articular damage	4 (1.2%)
Metabolism	25 (7.7%)
Glucose metabolism, proteolysis, peptide or amino acid transporter and protein metabolism, lipid metabolism and other metabolic processes	
Signal transduction and signaling pathways	16 (5.0%)
Phosphorylation and dephosphorylation, kinase activity and regulation, signal transduction and regulation, G protein-coupled receptor signaling pathway and others	
Tumor- and disease-related	8 (2.5%)
Neuron development, neurotransmitters and neuropeptide signaling	4 (1.2%)
Ion binding and transporters, binding activity	7 (2.1%)
Undefined function and unnamed genes	59 (18.3%)

^a^DEG: differentially expressed gene; tRNA: transfer RNA. "Immune activity" group includes DEGs in the first three categories: 50 (15.5%).

**Table 2:** Summary of differentially expressed genes in the lymph node cells of different groups of Lewis rats tested *ex vivo *and *in vitro*^a^

	DEGs, *n *(%)		
Characteristics	Upregulation	Downregulation	Total
*Ex vivo*			
Inc/Nv	322 (100%)	0 (0%)	322
Pk/Nv	31 (53.4%)	27 (46.6%)	58
Rec/Nv	28 (80%)	7 (20%)	35
*In vitro*			
Preclinical arthritis rats	41 (67.2%)	20 (32.8%)	61
Preclinical Bhsp65-tolerized rats	579 (98%)	12 (2%)	591

^a^DEG: differentially expressed gene. "*Ex vivo*" group shows DEGs in lymph node cells (LNCs) at incubation (Inc), peak (Pk) or recovery (Rec) phase compared to naïve (Nv) state; "*in vitro*" group shows DEGs induced by mycobacterial heat shock protein 65 (Bhsp65) compared to baseline control (LNCs cultured in medium alone). In Bhsp65-tolerized group, only 76 (12.86%) of the 579 DEGs were related to immune activity (innate immunity, cell-mediated immunity and humoral immunity). The major groups comprised genes relating to cellular proliferation 127 (21.5%) and metabolism 137 (23.2%).

To monitor the progression of the disease after the onset of AA, we analyzed genes that were differentially expressed in LNCs at the time of acute disease (Pk) and during recovery from acute arthritis (Rec), with each phase compared to that of the Nv rats. Both Pk and Rec phases of AA were associated with the expression of a relatively small number of genes (Figures 1, 2A and 2C). In the Pk phase, 31 genes were upregulated but 27 were downregulated, whereas in the Rec phase, 28 genes showed increased expression but 7 displayed reduced expression.

The relationship of the genes expressed at different phases of AA is shown in a dendrogram derived from cluster analysis (Figure 2B) and in a Venn diagram (Figure 2C). As depicted in Figure 2C, only 15 genes (*Cd163, Klrc1, Lgmn, Tnfrsf4, Il1r2, Ifitm1, Il23r, Ccr4, Cpd, Lipg, Rarres1, Olfm1, Mt1a *and two undefined genes) each were differentially upregulated in all three phases of the disease (Inc, Pk and Rec); 23 genes each were differentially expressed both at the Inc phase and during the Pk phase; 16 genes each were active in both the Pk and Rec phases; and 18 genes each shared a common expression pattern in the Inc and Rec phases of AA.

Since a large number of DEGs were revealed at the early preclinical phase (Inc), which is devoid of any clinical signs of arthritis, we propose that these genes are of significance in the initiation and subsequent progression of AA. To gain insight into the biological processes that might be influenced by DEGs in the Inc phase, we assigned the 322 early genes to separate groups according to their corresponding protein function and Gene Ontology classification (Table 1). We found that the DEGs at the Inc phase included the genes encoding the proteins related to cell proliferation, immune activity, inflammation, cell migration (including chemokines, chemotaxis and cell adhesion) and proteolysis, as well as certain metabolic and signal pathways.

To validate our microarray findings at different phase of AA, we performed qPCR on a set of randomly selected genes among those relevant to arthritis: *IFN-γ, IL-10, IL-17, Nos2, CCR5, Socs1 *and *Socs3*. The Bland-Altman plots (Figure 2D) suggest that all expression levels are within the 95% confidence limits for agreement, suggesting reasonable agreement of the expression obtained with the two methods.

As the Inc phase of AA revealed the most marked differences in gene expression, we chose this phase to further study the gene expression profiles of Bhsp65-restimulated LNCs of Mtb-immunized LEW rats and Bhsp65-tolerized, Mtb-immunized LEW rats.

### Antigen (mycobacterial heat shock protein 65)-induced gene expression profile of LEW rats in the preclinical phase of adjuvant arthritis

**Figure 3:**
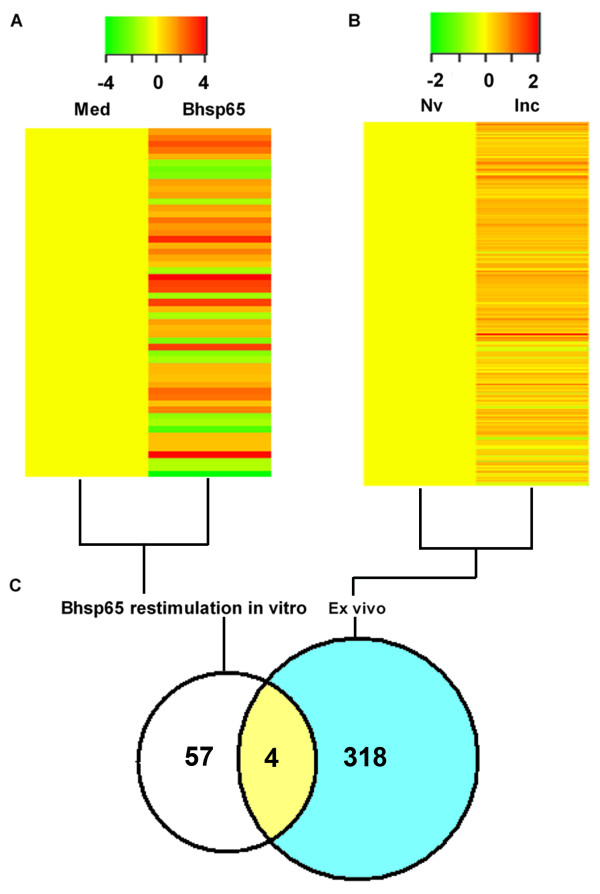
**Comparative gene expression profiles of antigen-stimulated lymph node cells *in vitro *and unstimulated lymph node cells *ex vivo *in preclinical arthritis rats**. A heat map representation of the DEGs of LNCs of different groups of rats is shown. **(A) **LNCs harvested from Lewis (LEW) (RT.1^l^) rats on day 7 after heat-killed *Mycobacterium tuberculosis *H37Ra (Mtb) injection (preclinical AA) were cultured *in vitro *for 24 hours with or without mycobacterial heat shock protein 65 (Bhsp65). **(B) **LNCs harvested from naïve (Nv) rats and from rats with preclinical arthritis (Inc) were tested *ex vivo *without culture *in vitro*. The results were compared as indicated in the figure. **(B) **Part of Figure 1 included for easy comparison with **(A)**. Red represents upregulated expression, and green indicates downregulated expression. **(C) **Venn diagram showing the relationship of DEGs in LNCs tested *ex vivo *versus Bhsp65-restimulated LNCs. DEG: differentially expressed gene; LNC: lymph node cell; Med: medium.

The precise autoantigen that induces immune disorder in RA remains unknown. Bhsp65 represents an important disease-related antigen in arthritis [39,40]. Several studies have revealed that rats with AA [7,12,27,40,41] and patients with RA [40,42-47] develop T-cell responses as well as antibody responses to heat shock protein 65. Furthermore, preventive or therapeutic interventions that suppress AA also alter immune responses to Bhsp65 [22,40,48]. In this context, we examined the expression profile of Bhsp65-induced genes in the draining LNCs of LEW rats in the Inc phase of AA. The LNCs harvested from LEW rats on day 7 after Mtb immunization were cultured for 24 hours with or without Bhsp65. The total RNA isolated from these LNCs was subjected to microarray analysis. The results are shown in Figures 3A, C and 5A. A total of 61 DEGs (41 upregulated and 20 downregulated) were found to be significantly influenced by Bhsp65. These genes showing altered expression encoded the leukocyte-specific markers and receptors, cytokines and receptors, chemokines and receptors, adhesion molecules, components of the complement cascade, molecules involved in antigen processing and presentation, regulators of angiogenesis, transcription factors and signal transduction-related molecules (Tables 2 and 3). Not surprisingly, the Bhsp65-induced gene expression profile mostly reinforced the immune-based and inflammatory nature of AA. The expression levels of important arthritis-related genes in these preclinical arthritis rats are given in Table 3.

**Table 3:** The comparative gene expression profiles of preclinical arthritis rats and Bhsp65-tolerized rats for the subsets of genes that play a role in the pathogenesis of arthritis^a^

		Fold change (compared to baseline)	
Gene symbol	Gene name	Preclinical arthritis rats	Bhsp65-tolerized rats
Costimulatory molecule			
*Cd86*	CD86 molecule	#	+2.06*
Cytokine or cytokine receptor			
*Ifi47*	Interferon γ-inducible protein 47	+2.44*	+2.37*
*Ifi27l1*	Interferon α-inducible protein 27-like 1	+2.14*	#
*Il1a*	Interleukin 1α	+4.61*	+3.21*
*Il1b*	Interleukin 1β	#	+3.05*
*Lta*	Lymphotoxin α (TNF superfamily, member 1)	#	+2.10*
*Socs1*	Suppressor of cytokine signaling 1	+3.67*	+3.14*
*Socs3*	Suppressor of cytokine signaling 3	#	+2.44*
*Ifng*	Interferon γ	+10.00*	+9.09*
*Il12rb2*	Interleukin 12 receptor β2	+4.08*	+3.89*
*Il10*	Interleukin 10	#	+2.07*
*Il33*	Interleukin 33	-2.50*	#
*LOC301289*	Similar to interleukin 17 precursor (IL-17)	+16.74*	#
*Il17f*	Interleukin 17F	+7.86*	#
*RGD1561292*	Interleukin 22	+7.26*	#
Chemokine/receptor			
*Cxcl10*	Chemokine (C-X-C motif) ligand 10	+8.02*	+8.15*
*Ccr5*	Chemokine (C-C motif) receptor 5	+2.22*	+2.50*
*Cxcr7*	Chemokine (C-X-C motif) receptor 7	-2.47*	#
Angiogenesis			
*Wars*	Tryptophanyl RNA synthetase	+2.29*	+2.17*
*Vegfa*	Vascular endothelial growth factor A	#	+2.21*
*Nos2*	Nitric oxide synthase 2, inducible	+7.83*	+4.77*
Others			
*Bst2*	Bone marrow stromal cell antigen 2	+2.90*	+3.04*
*Slc7a2*	Solute carrier family 7 (cationic amino acid transport)	+4.98*	+4.15*

^a^Bhsp65: mycobacterial heat shock protein 65. The genes listed were selected using false discovery rate < 5% and ≥2-fold change as the cutoff; +, upregulation; -, downregulation (- number); *significant compared to the corresponding baseline expression; #not significant compared to the corresponding baseline expression.

### Gene expression profile of mycobacterial heat shock protein 65-tolerized LEW rats and its comparison with that of LEW rats in the preclinical phase of adjuvant arthritis

**Figure 4:**
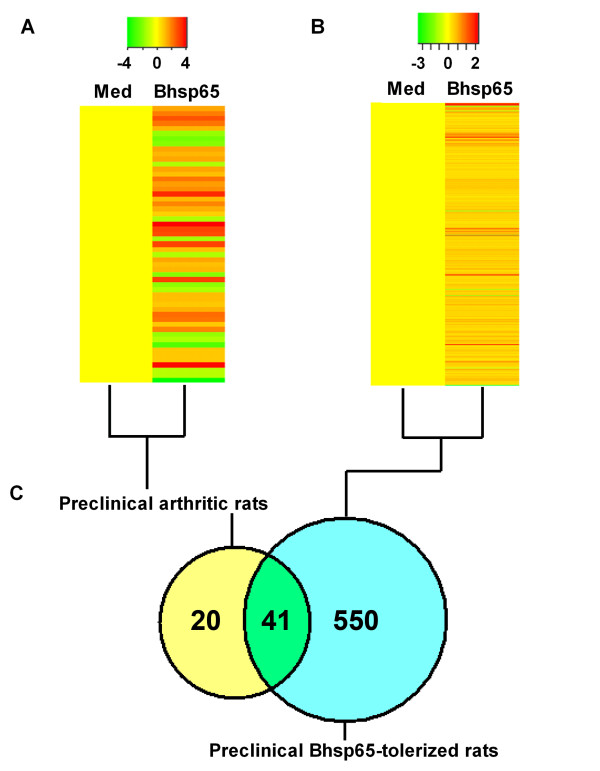
**Comparative gene expression profiles of antigen-stimulated lymph node cells of mycobacterial heat shock protein 65-tolerized rats versus preclinical arthritis rats**. A heat map representation of the DEGs induced by mycobacterial heat shock protein 65 (Bhsp65) in LNCs is shown. **(A) **LNCs harvested from Lewis (LEW) (RT.1^l^) rats on day 7 after heat-killed *Mycobacterium tuberculosis *H37Ra (Mtb) injection (preclinical AA) were cultured *in vitro *for 24 hours with or without Bhsp65. **(B) **LNCs of Bhsp65-tolerized rats were processed in the same manner. The results were compared as indicated. **(A) **represents part of Figure 3 and is included for easy comparison with **(B)**. Red represents upregulated expression, and green indicates downregulated expression. **(C) **The number of overlapping DEGs in different groups of rat LNCs tested. AA: adjuvant arthritis; DEG: differentially expressed gene; LNC: lymph node cell; Med: medium.

**Figure 5:**
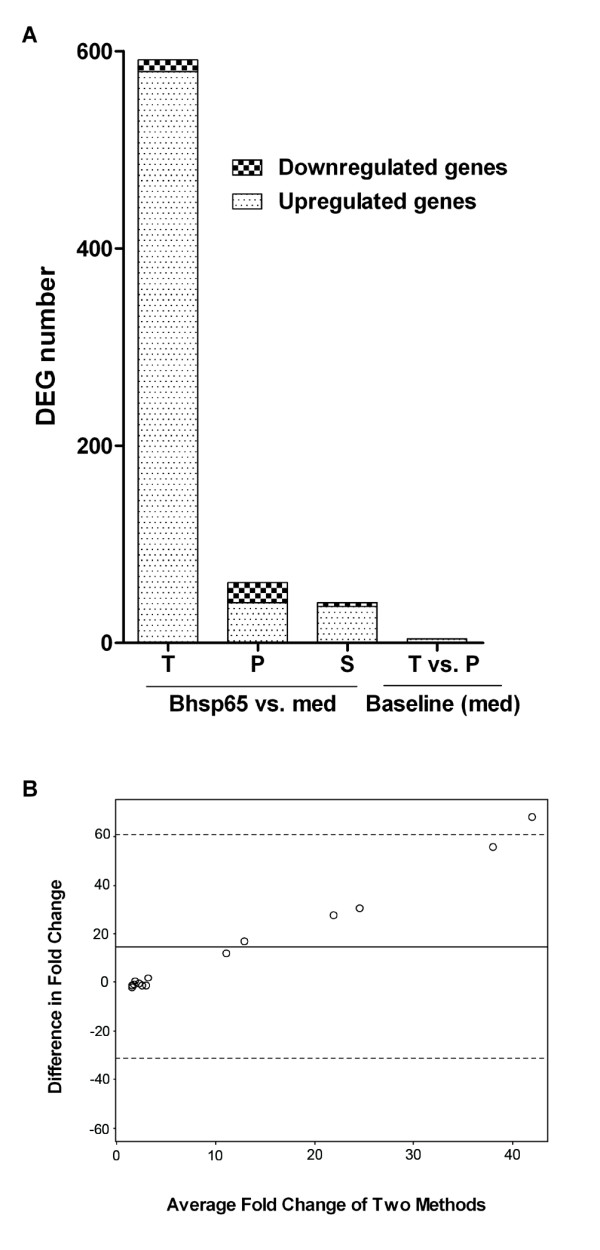
**Comparison of the gene expression patterns of mycobacterial heat shock protein 65-tolerized rats and rats with preclinical adjuvant arthritis**. **(A) **DEGs in LNCs of mycobacterial heat shock protein 65 (Bhsp65)-tolerized rats (T) and rats with preclinical AA (P) are shown. Also shown are DEGs shared (S) between the two groups (data derived from Figure 4). **(B) **Bland-Altman plots. The *x*-axis represents the average fold change of each sample measured by quantitative real-time PCR (qPCR) and microarrays. The *y*-axis is the difference in fold change calculated by qPCR measurement minus microarray measurements for each sample. The solid line (*y *= 14.89) is the mean difference in fold change of all the samples. The two dotted lines represent two standard deviations from the mean. AA: adjuvant arthritis; DEG: differentially expressed gene; LNC: lymph node cell; med: medium.

We described above that Bhsp65 represents an important disease-related antigen in LEW rats with AA [7,12,27,40,41]. Accordingly, Bhsp65 also offers an attractive antigen for use in the immunomodulation of AA [7,10-12]. In fact, induction of immune tolerance against Bhsp65 can successfully downmodulate the onset and progression of AA [22]. However, the mechanisms involved are not fully defined. To identify the genes that might be involved in the modulation of AA by Bhsp65-induced tolerance, and also to identify additional potential autoimmune targets for therapy, we compared the mRNA expression profile of LNCs from Bhsp65-pretreated, Mtb-injected LEW rats after seven days of disease induction (Figure 4B) with that of the Mtb-immunized LEW rats in the Inc phase of AA (Figure 4A). For each group of rats, we compared the profile of LNCs restimulated by Bhsp65 *in vitro *with that of LNCs cultured in medium alone (baseline level). The Bland-Altman plots (Figure 5B) suggest that all expression levels are within the 95% confidence limits for agreement, suggesting reasonable agreement of the expression levels obtained with the two methods.

Although the baseline level of gene expression (in LNCs in medium alone) in Bhsp65-tolerized LEW rats and LEW rats with preclinical AA showed little difference (four DEGs only), there were substantial differences in DEGs (Bhsp65 restimulation vs. medium *in vitro*) in Bhsp65-restimulated LNCs of these two groups (Figures 4C and 5A). The total DEGs numbered 591 for the Bhsp65-tolerized group compared to 61 for the preclinical AA group. Furthermore, the upregulated genes comprised 98% (579 of 591 genes) of DEGs in the Bhsp65-tolerized rats (Figures 4B and 5A) but only 67.2% (41 of 61 genes) of DEGs in the rats with preclinical AA (Figures 4A and 5A). Interestingly, the upregulated DEGs in Bhsp65-tolerized rats reflect a spectrum of immune markers and pathways, including T-cell costimulatory molecule, cytokines and receptors, chemokines and receptors, and angiogenesis (Tables 2 and 3). In comparison with preclinical arthritis rats, Bhsp65-tolerized rats showed downregulation of Th1 and Th17 (proinflammatory response) and of other mediators of inflammation and angiogenesis, but upregulation of IL-10 (anti-inflammatory and immunoregulatory). At least 12 arthritis-related DEGs constitute the molecular signature of Bhsp65-induced tolerance (Table 3). These genes encode the following proteins: CD86, IFN-α-inducible protein 27- like 1, IL-1β, lymphotoxin-α, SOCS3, IL-10, IL-33, IL-17 precursor, IL-17F, IL-22, CXCR7 and VEGF-A.

Antigen-induced tolerance is generally perceived to be a downmodulatory effector response in which activated immune system events are suppressed. Accordingly, it is presumed that the levels of expression of several genes associated with immune effector pathways would be downregulated in Bhsp65-tolerized rats compared to preclinical arthritis rats. In this context, our results showing that the number of genes with upregulated expression levels is much higher in Bhsp65-tolerized rats than those in preclinical arthritis rats (Tables 2 and 3) indicate that the state of immune tolerance is an active process involving enhanced gene expression. We interpret this as activation of those immune pathways that can induce attenuation of pathogenic immune responses. For example, enhanced expression of genes encoding the proteins involved in immunoregulatory activities (for example, IL-10) might explain the observed profile of gene expression.

## Discussion

On the basis of using the rat adjuvant-induced arthritis model of human RA and microarray technology in this study, we describe the gene expression profiles of arthritic LEW rats at different phases of the disease as well as the modulation of gene expression by a tolerogenic disease-protective regimen employing the disease-related antigen Bhsp65. We tested the draining LNCs of arthritic rats *ex vivo *as well as after their restimulation with Bhsp65. We extended this analysis to the LNCs of LEW rats administered a tolerogenic challenge of Bhsp65, which results in a significant reduction in the severity of arthritis [22]. The criteria for a positive gene expression response (for example, FDR set < 5% and fold increase) are outlined in the Materials and methods section.

### Gene expression profiles during the natural course of adjuvant arthritis in LEW rats

The natural course of AA in LEW rats is discernible in distinct phases: Inc, Pk and Rec. We compared the *ex vivo *gene expression profile of LNCs at each of these phases with that of Nv LEW rats, which served as the baseline. Our results reveal that the maximum changes in gene expression, both quantitatively and qualitatively, were observed at the Inc phase of arthritis instead of the Pk phase of the disease. In fact, most of the genes showed significantly reduced expression at the Pk and Rec phases compared to the Inc phase. As the LNCs were tested *ex vivo *directly after being harvested from the rats, the observed patterns of gene expression likely represent the natural *in vivo *expression profiles. These results show that the Inc phase of AA is a critical and very active stage of the disease in terms of changes in the expression of genes encoding a large number of proteins that participate in the induction of arthritis. The Inc phase of AA is equivalent to the preclinical phase of human RA. Therefore, our results are of significance in advancing our understanding of the initiation of the disease process. Furthermore, as yet there is no reliable biomarker that can predict the induction of RA in a given individual. On the basis of our results described above, we are hopeful that similar studies in RA patients might lead us to discover the much-needed biomarkers of diagnostic and prognostic value. In addition, as described below, such analysis would also be of great utility in defining the molecular changes induced by an immunomodulatory (preventive) regimen for arthritis.

The DEGs at the Inc phase were related to cell proliferation, immune activity, inflammation, cell migration (including chemokines, chemotaxis and cell adhesion) and proteolysis (Table 1). The most abundantly represented genes were those associated with cell proliferation (113 genes, 35%); however, barely any of these genes were found to be expressed in the later phases of AA. The immune activity genes, including both innate and adaptive immune responses, were highly represented at the early phase (Inc; 42 genes, 13%), but were much less abundant at the Pk phase (19 genes) and the Rec phase (10 genes). The immune activity genes with a significant change in expression levels included those encoding the immune cell markers CD14, CD163 and CD163l1; Th1 and Th17 cytokines and cytokine receptors; immunoglobulins; and complement components. All of these are relevant for promoting inflammation and immune damage. Also, upregulated were the genes for IL-1 receptor type II (Il1r2), IL-1 receptor antagonist (Il1ra) and suppressor of cytokine signaling 3 (Socs3). The increased expression of some of the anti-inflammatory genes, along with the enhanced expression of many proinflammatory genes, most likely reflects the attempt of the host to counter the emerging inflammation.

The infiltration of inflammatory cells into the joints is believed to initiate the activation of synovial cells and subsequent hyperplasia of the synovial lining, which eventually leads to destruction of the cartilage and bone in arthritic joints [3,49,50]. Therefore, the migration of immune cells into the joints is a critical trigger for disease induction in arthritis. We found altered expression of 24 genes (7.5%) that facilitated cell migration at the Inc phase, but only 3 at Pk and Rec phases combined. These results suggest that cell migration into the joints is facilitated in the Inc period, which then triggers the inflammatory events evident at the onset of AA. In addition, surprisingly, the numbers of genes encoding the extracellular matrix degradation-related proteins that are relevant to bone destruction are more abundant in the early (Inc) phase compared to the Pk and Rec phases of AA. These genes encode latexin, matrix metallopeptidase 14 (Mmp14) and membrane metalloendopeptidase. *Mmp8*, another important gene involved in the pathogenesis of bone damage, was significantly upregulated in the Pk through Rec phases.

Recent studies examining the role of oxygen metabolism in the pathogenesis of arthritis have revealed various inflammatory mediators linked to the destruction of joint tissue. In our study, of 322 DEGs in the early phase of AA, 11 genes (3.4%) related to oxygen metabolism were upregulated. Three genes encoding different hemoglobin components were found to be downregulated at the Pk phase, and these genes might be associated with severe hypoxia. However, no DEGs related to oxygen metabolism were found in the Rec phase. We found that the S100 family members S100A4 (S100a4), S100A9 (S100a9) and S100A11 (S100a11) were upregulated before the signs of arthritis appeared. Two members of this family, S100A8 and S100A9, are particularly susceptible to oxidative modification [51]. These two proteins, which are abundantly expressed in neutrophils and activated macrophages, are associated with various inflammatory conditions, including RA [51].

As described above, 15 genes were upregulated throughout the course of AA. In view of the function of these 15 genes, it was evident that multiple cellular and biological processes are involved in the progression of AA. CD163 is expressed in monocytes and macrophages and subsets of dendritic cells, which play an important role in the pathogenesis of AA. Costimulatory signals via *Tnfrsf4 *(CD134) and antigen presentation via the major histocompatibility complex class II pathway facilitated by the enzyme legumain (encoded by *Lgmn*) represent additional important events in the development of AA. TNF and IFN (as inferred from the expression of *Ifitm1*) are proinflammatory cytokines that are known to play a pathogenic role in AA. Additionally, the Th17 response (inferred indirectly from the sustained expression of IL-23R) is another vital event in the disease process in AA. The progression of AA also involves migration (indicated by *Ccr4 *expression) of inflammatory cells into the target organ, the joint.

### Mycobacterial heat shock protein 65-induced gene expression profile of LEW rats in the preclinical phase of adjuvant arthritis

The analysis of the Bhsp65-induced gene expression profile of rats in the Inc phase of AA mostly reinforced the immune-based and inflammatory nature of AA. Among the upregulated genes, 56% (23 of 41) were relevant to immune activation, and almost half of them were genes related to cytokine-cytokine receptor interactions. Upon detailed examination, we observed increased expression of *Il1a *(4-fold); Th1-related cytokine and cytokine receptor or transcription factor, including *Ifng *(10-fold), *Il12rb2 *(4-fold), *Tbx21 *(2.6-fold) and *Stat1 *(1.8-fold); Th17-related genes, including *CTLA-8 *(17-fold) and *Il17f *(8-fold); and IL-22 precursor (7-fold). A notable exception was *IL-33*, whose expression was reduced by 60%. In addition, the expression of B-cell-cycle-activated gene *Inhba *and complement gene *Cfb *was increased. The genes pertaining to chemokines and their receptors (for example, *Cxcl10 *and *Ccr5*) were also represented in the list of upregulated genes. The expression of chemokines and their receptors plays a critical role in regulating cell trafficking and other inflammation-related events. The *Cxcl10 *gene showed an eightfold increase in expression. CXCL10, which is one of the ligands for CXCR3, is an IFN-γ-induced small protein secreted by cells in response to IFN-γ. CXCL10 is chemotactic for monocytes, macrophages, neutrophils, T cells, natural killer cells and immature dendritic cells [52] and is also involved in promoting T-cell adhesion to endothelial cells [53]. Of interest, it has been reported that CXCL10 can be detected at high levels in synovial tissue [50] as well as the synovial fibroblast cell lines derived from RA patients [54]. Furthermore, CXCR3 and its ligands are involved in the selective recruitment of Th1 effector cells into the sites of tissue inflammation [55,56]. The gene for the receptor for another chemokine, CCR5, was also upregulated after Bhsp65 restimulation. CCR5 is preferentially expressed on Th1 cells, and CCR5-expressing cells are enriched in the affected joints of RA patients [49]. Taken together, altered expression of the genes related to Th1 and Th17 responses is the most predominant change following Bhsp65 restimulation of LNCs of preclinical arthritis rats.

A major difference was observed in the expression of the cytokine genes. Bhsp65-restimulated LNCs revealed changes in multiple cytokine genes (12 of 61 genes, 19.7%) that showed a high level of expression, in contrast to only four cytokine genes (4 of 322 genes, 1.24%) that showed increased expression in the LNCs of Mtb-immunized rats tested *ex vivo *without any Bhsp65 restimulation. The observed differences in DEGs between Mtb-stimulated LNCs tested *ex vivo *and Bhsp65-restimulated LNCs *in vitro *might be attributable to the restimulation of a specific set of genes following reexposure *in vitro *to Bhsp65 from among the genes whose expression was influenced by immunization with Mtb, which contains multiple antigens.

### Gene expression profile of mycobacterial heat shock protein 65-tolerized LEW rats

We have previously shown that the treatment of LEW rats with soluble Bhsp65 delivered intraperitoneally led to the induction of antigen-specific tolerance as well as significant reduction in the severity of AA [22]. In this context, we reasoned that the disease-protective effect of tolerization with Bhsp65 might involve significant downregulation of the expression of genes pertaining to multiple pathways. However, the results of our experiments presented an intriguing and opposite picture in that a large number of Bhsp65-inducible genes were rather upregulated in Bhsp65-tolerized rats compared to the control preclinical arthritis rats. These results show that antigen-induced tolerance is an active process that upregulates a variety of genes instead of a process that mostly downregulates gene expression (Table 3). This finding is contrary to the general impression that tolerogenic regimens typically shut down immune events. Understandably, the immune activation processes during tolerance induction would target pathways that facilitate regression of inflammatory arthritis, which would explain the disease-protective effects of the tolerogenic regimen.

Most of the DEGs (41 of 61, 67.2%) in rats with preclinical AA were also represented among the DEGs of Bhsp65-tolerized rats (Figure 4C). More interesting than the higher number of DEGs in Bhsp65-tolerized rats is the relationship of the selectively upregulated or downregulated genes to various disease-related processes in AA. For example, among the immune response-related genes, those encoding Th2 response-related molecules, such as IL-10, IL-33 and IL-15 receptor α chain (IL-15Rα), were upregulated in Bhsp65-tolerized rats, but those for IL-10 and IL-15Rα were unaltered in preclinical LEW rats. These results show that the anti-inflammatory cytokines play a vital role in the regulation of arthritis following Bhsp65-induced tolerance, with a shift of the T-cell phenotype response to anti-inflammatory (Th2) type. Furthermore, no Th17 response-related genes were upregulated in Bhsp65-tolerized rats, which is supported by the results of our previous study showing a significant reduction in IL-17 in Bhsp65-tolerized vs. control rats [22]. These results show that the regulation of arthritis by soluble Bhsp65-induced tolerance involves comprehensive interactions among different immune molecules. The increased expression of cell cycle-related genes in tolerized rats might reflect a rapid activation of immune cells followed by cell apoptosis, which then interferes with further immune stimulation after Mtb immunization. We propose that the testing of gene expression profiles at the Inc (preclinical) phase of arthritis might help define the mode of action of the disease-protective regimen for arthritis by using antigens, synthetic drugs or natural products [9,22].

As elaborated above, our results derived by microarray analysis have revealed significant changes in a large number of genes representing proteins that participate in multiple pathways, including various immunological and biochemical pathways (Table 1). At best, microarray analysis can reveal transcriptional changes in the genes encoding functional proteins. As the processes of transcription and translation of mRNA are controlled at multiple levels, and since the final products (the encoded proteins) can be further modified by posttranslational modifications, it is likely that some of the extrapolations based on mRNA expression might not materialize at the final protein level. Also, many of the transcripts on the gene chip used in our study have not yet been identified. Therefore, a follow-up study of protein expression profiles in AA is needed to confirm the extent of the changes implied by our microarray analysis.

As described above, in this study, we examined the gene expression profile of the draining LNCs of arthritic rats and rats subjected to antigen (Bhsp65)-induced tolerance. A major proportion of the upregulated genes were relevant to immune activation (Tables 1 and 3) and include the genes related to cytokines and cytokine receptors, cell migration (adhesion molecules, chemokines and chemokine receptors), angiogenesis and articular damage. Interestingly, most of the genes identified in the LNCs are also relevant to the arthritis-related events in the periphery and the target organ, the joints. The T cells reactive against mycobacterial antigens can be detected in the spleen, peripheral blood, synovial fluid and synovial tissue of arthritic animals [7,10,40,41] as well as in patients with RA [42-44,46,47]. Similarly, proinflammatory cytokines (for example, IFN-γ, TNF-α and IL-17) can be detected in these body fluids, cells and tissues [57-59]. Importantly, some of these genes and proteins can serve as biomarkers (for example, TNF-α and IL-17) for disease monitoring. Similarly, chemokines and their receptors, such as CCR5, CXCR7 and CXCL10, can be detected in the leukocytes infiltrating the synovial tissue in arthritic rats and RA patients [49,50,52,54,60-62]. In addition, the process of neoangiogenesis driven by vascular endothelial growth factor (VEGF) is a hallmark of arthritis in experimental animals and RA patients [63,64]. These observations validate the significance of our results obtained by testing LNCs.

## Conclusions

Our study is the first to report the gene expression profile of AA. We believe that, together with previous reports of microarray analysis in other experimental models of arthritis [65-70], the results of our study significantly advance our understanding of the pathogenesis of autoimmune arthritis. In particular, by revealing that the maximal changes in gene expression during the natural course of AA occur in the preclinical (incubation phase) of the disease, we have highlighted the significance of the preclinical phase of arthritis for further defining the immunopathogenic events in arthritis as well as for studying the impact of an immunomodulatory regimen such as antigen-induced tolerance. We have identified a molecular signature consisting of at least 12 arthritis-related genes whose expression was modulated significantly following Bhsp65-induced tolerance (Table 3). These genes encode for CD86, IFN-α-inducible protein 27- like 1, IL-1β, lymphotoxin-α, SOCS3, IL-10, IL-33, IL-17 precursor, IL-17F, IL-22, CXCR7 and VEGF-A. We believe that the results of these above-referenced microarray-based studies in animal models [65-70] and limited studies in RA patients [68,71,72] provide a strong foundation for further studies of the preclinical phase of RA and for identifying reliable biomarkers for the diagnosis, prognosis and therapeutic evaluation of this debilitating disease.

## Abbreviations

AA: adjuvant arthritis; Bhsp65: mycobacterial heat shock protein 65; DEG: differentially expressed gene; IFN: interferon; Inc: incubation; IL: interleukin; LEW: Lewis; LNC: lymph node cell; Med: medium; Mtb: *Mycobacterium tuberculosis *H37Ra; Nv: naïve; qPCR: quantitative real-time PCR; RA: rheumatoid arthritis; Pk: peak; Rec: recovery; TNF: tumor necrosis factor.

## Competing interests

The authors declare that they have no competing interests.

## Authors' contributions

HY performed the experiments, analyzed and interpreted the data, prepared the figures and tables, wrote the manuscript and helped in designing the experiments. CL and MTT performed data analysis and helped in writing part of the Materials and methods section. KDM designed the study, interpreted the data and wrote the manuscript. All authors read and approved the final manuscript for publication.
